# Epithelial Cancer of the Tongue

**Published:** 1874-12

**Authors:** 


### AMERICAN JOURNAL

OF

### DENTAL SCIENCE.

Vol. VIII. THIRD SERIES--DECEMBER, 1874. No. 8.

### ARTICLE I.

### *Epithelial Cancer of the Tongue.*

BY DR. HUBBARD.

Read before the California State Dental Association.

The definition Woods gives of the subject announced above is, that "it is that kind of cancer which assumes the form of an irregular and exuberant deposit of cells identical with, or at least resembling closely, those cells which form the normal covering or investment of mucous membrane and skin, and which are called 'epithelial,' and are, so to speak, formations germain to the parts affected." The common forms of disease are included by Hanover, under the name *ephithelioma.*

It is considered by the best pathologists I have been able to consult, to resemble so closely in its course and effects the real carcinomatous diseases of scirrhus and cephaloma, as to entitle it to the fatal distinction of being placed among the cancerous diseases; while others consider it to be cancroid, or resembling it in its characters. The most characteristic features are its uncontrolable growth; its progressive invasion of neighboring tissue; its supplanting or pushing aside and destroying the growth of the normal structures; the very severe darting pains which attend its course, caused by its involving the ultimate fibrels of the nerves of the part, in the same manner perhaps as a corn affects them under the pressure of the boot; its liability to invade the lymphatics, and so to make its way into the system; and its occasional recurrence in the deeper organs and viscera, sometimes as ephithelial deposits, and sometimes as one of the more decided malignant growths, namely "encephaloma."

On the other, hand it is more tardy in its fatal progress than the more virulent forms of carcinoma, and is much less apt to recur in the deep seated viscera as a secondary deposit, though its progress resembles in many important points that of scirrhus or hard cancer, especially that aspect of the latter, in which the advocates of its local origin and slow continuously progressive course view it; and sometimes its microscopic elements can, taken by themselves, with difficulty be distinguished from some of the cell forms of scirrhus cancer.

This is especially the case in that form of indigenous cell, designated by Pagot as "broad cells," where more than one nucleus is seen enclosed in a mother cell, as is usually the case in hard cancer. In some of its more mild manifestations, epithelial cancer can scarcely be distinguished from an ordinary warty growth, such as that which is common enough on the hands of young persons. In both may be observed the masses of clustered, more or less flattened, epithelial cells, lying upon each other concentrically, like the layers of an onion, with little or no fibrous or connective tissue, and forming by the intimate adhesion of the cells themselves a very tough, hard resisting mass. It is only by the persistency and recurrence of the growth, the pain which attends it, its gradually spreading beyond the site originally affected, and its effects upon the general health that epithelial cancer can be distinctly diagnosed. In many cases the disease is said to become developed out of so called benign warty tumors, which are consequently usually suspiciously viewed by practical surgeons. And it may be said that the more closely adherent, hard and slow growing the tumor, the less disposed it is to ulcerate, and the less painful in its manifestations, the less dangerous its character may be estimated to be; while conversely, the more loosely its cells are held together, and the more disposed it is to become disintegrated and to form an ulcer, the more rapid is its course, and the sooner do the lymphatic glands become affected. I find some cases recorded in the "Transactions of the Pathological Society of King's College Hospital," (to which I was allowed access by courtesy of G. G. Tyrrell, M. D., F. R. S.,) in which the amount of fibrous stroma produced by the development of fibre-cells in the mass is so great that the disease assumes a more doubtful pathological character, and may be assigned to another place in the classification of pathologists, more closely approaching to the scirrhus form of carcinoma. Dr. Clark, in his monograph on the diseases of the tongue, states that he believes some cases of so called scirrhus, to have been of this kind. Again, the disintegration of the cell growth may be so rapid that the features of the disease may assume the form of an open ulceration of a malignant character rather than that of a tumor; and many such cases I doubt not have been denominated "Cancerous Phagedæna." In conclusion then, I would say, that as a general thing the more advanced and minute pathological observations become, the less disposed are practical men to draw any broad line of demarcation between these diseases. In well marked cases the signs are plain enough; but at a time when diagnosis is most valuable, the indications are usually the most slight.

To remove the disease, the least painful, most manageable, and most effective plan is no doubt by the application of the knife. Caustics in this situation work too slowly and superficially, and too painfully and uncontrollably to be valuable in any other way than as secondary to the absolute removal of the diseased mass. Used in this way, they are of great value, and were employed by me in the cases I have had in the form of a solid or saturated solution of the chloride of zinc, sometimes combined with the perchloride of iron, and frequently applied to destroy any remains of the disease which may have escaped the knife. The latter has also the additional advantage of stopping any bleeding that may still further lower the resisting powers of the patient to the invasion of the disease.

In most of the books I have been able to find, the operations for the removal of the tongue, or any part of it, has been considered as one of those in which the blessings of anæsthesia could not with safety be afforded to the patient, for the reasons that will be at once apparent, viz: first, the removal by the anæsthetic agent of the voluntary assistance which the patient can render in all operations upon the interior of the mouth, by keeping the jaws and mouth wide open and the tongue steady; and secondly, the supposed danger in profuse bleeding into the pharynx of suffocation, by the blood passing into the larynx and trachea. It is very evident that if we cannot in these cases offer to our patient a means of relief, unattended by immediate pain, and that of a most severe character, at least half of the advantages of and inducements to the treatment involving such an operation are thrown away, and the patients will be naturally indisposed to listen to the only promise of relief which can honestly be held out to them. If then we can render by any means the necessary operations painless, we confer, I think, a boon upon humanity not by any means small, to the poor creatures thus afflicted. After some little contriving I made a gag, which I think meets every case, and enables the surgeon to use anæsthetics with some degree of safety. It consists of four teeth plates, which act on both the upper and lower bicuspids by means of a pair of powerful cross levers, placed outside of the cheeks, and opening independently of each other by a screw on each side through a simple hinge joint. Having thus obtained the necessary control of the movements of the patient's lips and jaws, during the insensibility produced by chloroform, we have only to look to those precautions which are usually found most efficacious, such as turning the head down to one side and clearing the throat with the finger. If the knife is to be used, I have found it to be the best plan to pass two stout ligatures through the base of the tongue, by means of a curved needle—these ligatures are held by an assistant. By this means we have the tongue extended to its utmost limits, and by being widely placed they prevent the turning out of sound fleshy substance, which is very apt to be the case when the ordinary vulsellum forcep is used. If the tumor is likely to be very vascular, as may in some measure be divined from its appearance, I deem it prudent to pass a broad ligature across the canine arteries, under the base of the tongue, by means of a curved needle; this controls the hemorrhage until the cut ends of the vessels have been secured. The tumor is next seized by a pair of stout double toothed lioned forceps, and then a curved, sharp pointed vistoury is used to transfix the tongue close to its base, and to cut first antero-posteriorly, and then laterally across the vessels and nerves, until so much has been cut as will suffice to rapidly clear away the diseased mass. In securing the arteries, much steadiness on the part of the assistant is required. The muscular substance of the tongue is so friable, under the circumstances, that it is readily broken down by too much vigor in drawing the knot; while the depth at which the cut vessels lie from the surface necessitates dexterity in slipping the thread over the forceps, the points of which should be conical and taper quickly to the end. The proper tying of the vessels, and the prompt application of the tincture of the perchloride of iron and chloride of zinc, by a strip of lint tied round a stick, will render the use of the arterial cautery rarely necessary to subdue the hemorrhage. In conclusion to the pathology and cure of the above disease, I will give two cases which

I have had during the last year. In February, Albert Stanchfield came to my office for treatment; he had noticed pain and soreness in his tongue, so long as ten years ago. Thinking it was irritated by the molar teeth on that side, he had them extracted four years ago, although they were perfectly sound; twelve months before he noticed, for the first time, that it was rather swollen and hard. The pain becoming worse six weeks before I saw him, he began to emaciate, and the cancer got rapidly larger and more painful, and the ulceration spread further. On examination I found a tumor about the size of a small egg on the left border of the tongue, hallowed out in the middle by an extensive ulceration, covered with an ash colored slough, and surrounded by fungus raised edges, with a very fetid discharge—frequent hemorrhages had occurred from the middle of the sore. A sub-maxillary lymphatic gland could be felt, enlarged and hard on the same side. There was a copious and distressing flow of saliva, and the poor man suffered excruciating agony both night and day; the effects of loss of sleep and suffering speedily became visible in his rapid emaciation and weak accelerated pulse. On the 9th of February, under chloroform, and aided with the gag I have described, I removed two-thirds of the tongue, cutting clear of the tumor, and afterwards taking away considerable portions which appeared to me to be rather suspicious in *feel* and appearance. Free hemorrhage occurred from the numerous vessels, which was with difficulty controlled, the substance of the tongue being exceedingly friable, and giving away easily under the ligatures. The chloride of zinc and the perchloride of iron were freely applied, both separately and in combination. A blackened slough was formed all over the sore which,aft er some days, came off, showing a smooth healthy and regular healing surface. The sore rapidily healed after a few applications of the solid chloride of zinc, which was employed to smooth down all irregular granulations; and on the 2d of March, the man looking vastly improved in health, free from all pain, and able to eat and speak with more comfort than he had had since the disease appeared, was discharged with the sore almost healed, at his own request, in order to look after his business in the country. The second case I will bring to your notice, was a well marked and advanced specimen of the disease of the tongue under consideration ; it was sent to me by a Dr. in Auburn—a man aged forty five, a delicate man, evidently much worn by suffering and interference with the taking of food. On opening his mouth I observed a large solid tumor of the size of a hen's egg, embracing the whole of the left half of the tongue, commencing, as in all other cases, opposite the bicuspid and anterior molar teeth. The surface of the tumor was covered with a deep ulceration, with deep ragged edges, and deep sloughy-looking fissures, and exhaling a very offensive odor and putrid discharge. There was no affection of the glands apparent on careful examination ; but the patient complained of deafness and much pain in the ear of the same side, probably from implication of the gustatory and chorda tympani nerves. He had great mechanical difficulty in mastication and deglutition, and has at times lost much blood by hemorrhage from the ulcerated surface of the tumor ; pain, want of food, hemorrhage had reduced him to a distressing condition. The disease in this case commenced only in December, and pursued a much more rapid course than any other that I have been able to find any record of. It began at first as usual with a decayed tooth. As it was so near the annual meeting of this society, I thought if it were possible I would try and keep the case along, in order that we might get some clinical instructions from it, so I put him on a preliminary course of iodide of potass and tartrate of iron, touching it with solid chloride of zinc twice a day ; but as no relief resulted from this treatment, and the poor man was getting gradually thinner and more cachectic from the constant wearing pain day and night, and the interference with his power of masticating food, I dared to delay no longer, and I operated two weeks ago. Much relief followed this operation, which was attended with comparatively little hemorrhage. It is astonishing how rapidly the patient is recovering from this operation; freedom from pain, undisturbed rest at night, improved appetite, and revigorated digestive powers, account for this satisfactory result.

